# Insights into the Genetic Evolution of Duck Hepatitis A Virus in Egypt

**DOI:** 10.3390/ani11092741

**Published:** 2021-09-19

**Authors:** Mohammed A. Rohaim, Rania F. El Naggar, Mohammed A. AbdelSabour, Basem A. Ahmed, Mohamed M. Hamoud, Kawkab A. Ahmed, Osama K. Zahran, Muhammad Munir

**Affiliations:** 1 Department of Virology, Faculty of Veterinary Medicine, Cairo University, Giza 12211, Egypt; mohammed_abdelmohsen@cu.edu.eg (M.A.R.); basem-ahmed@cu.edu.eg (B.A.A.); 2 Department of Virology, Faculty of Veterinary Medicine, University of Sadat City, Sadat 32897, Egypt; rania.elnagar@vet.usc.edu.eg; 3 Department of Poultry Viral Vaccines, Veterinary Serum and Vaccine Research Institute (VSVRI), Agriculture Research Centre (ARC), Cairo 11381, Egypt; dr.m.adel.vsvri@gmail.com; 4 Department of Poultry and Rabbit Diseases, Faculty of Veterinary Medicine, Cairo University, Giza 12211, Egypt; mohamed.hamoud@cu.edu.eg; 5 Department of Pathology, Faculty of Veterinary Medicine, Cairo University, Giza 12211, Egypt; kawkababdelaziz@yahoo.com; 6 Department of Animal Hygiene and Veterinary Management, Faculty of Veterinary Medicine, Cairo University, Giza 12211, Egypt; zahran771953@gmail.com; 7 Division of Biomedical and Life Science, Lancaster University, Lancaster LA1 4YG, UK

**Keywords:** duck hepatitis virus, ducklings, genotype I, natural selection, vaccines

## Abstract

**Simple Summary:**

The accumulation of point mutations and/or recombination between different serotypes drives the evolution of the duck hepatitis A viruses (DHAVs). Duck viral hepatitis, caused by DHAV, is a highly infectious disease that has been detected in Egypt since 1970. Low performance, nervous signs and sudden deaths of young ducklings were the main characteristics of the disease. The aim of this study was to identify the causative agent through viral and molecular detection for the causative virus. The causative virus was isolated in embryonated duck eggs (EDEs), with complete genome sequencing that indicate the clustering of our isolates reported in this study with Chinese duck hepatitis virus 1 that may help in new vaccine manufacturing and the development of a more sensitive diagnostic assays. Future studies to evaluate the potential protection of available commercial vaccines against the newly detected isolates will be needed.

**Abstract:**

Duck hepatitis virus (DHV) is one of the commercially important diseases of ducklings worldwide. It is an acute and highly infectious disease of ducklings caused by three different serotypes (1–3) of duck hepatitis A virus (DHAV), and serotype 1 is the most common in poultry. To date, little is known about the prevalence and genetic characterisation of DHAV-1 in Egypt. In the current study, isolation and complete genomic analyses of DHAVs circulating in commercial duck farms in different Egyptian governorates were conducted. A total of eighteen samples were collected from six Egyptian governorates of 3–11 days old ducklings (Pekin and Mullard) with a history of nervous signs and high mortality rates. Five out of eighteen (5/18) samples were screened positive for the DHAV-1 based on the VP1 gene. These samples were individually used for virus isolation in embryonated duck embryos (EDE), followed by complete genome sequencing. Phylogenomic analyses showed that DHAV serotype I; genotype I were diversified into four different groups (1–4). Most of the recent circulating Egyptian DHAV strains are clustered within group 4, while isolates characterised within this study were clustered within group 1. Recombination analyses revealed that the emergence of a new recombinant virus—DHAV-1 strain Egypt-10/2019—through recombination. Likewise, the selective pressure analyses showed the existence, inside or near areas of the viral attachment or related functions, of positive scores highlighting the importance of natural selection and viral evolution mechanism at different protein domains. The findings of this study provide updated information on the epidemiological and genetic features of DHAV-1 strains and underscore the importance of DHAV surveillance as well as re-evaluation for currently used vaccines.

## 1. Introduction

Duck viral hepatitis (DVH) is a highly contagious disease of ducks characterised by severe morbidity and mortality, particularly in ducklings under the age of four weeks old [1]. Duck hepatitis A virus (DHAV), causative of DVH, is classified into three types I, II and III. The DHAV type I belongs to the genus *Avihepatovirus*, family *Picornaviridae* and has three distinguishable serotypes designated as serotypes 1–3. Meanwhile, DHAV types II and III were recently classified as duck astrovirus 1 and 2 (DAstV-I and DAstV-II) and belong to the family *Astroviridae* and are antigenically distinct from DHAV type I. DHAV serotype 1 is the most widespread serotype; however, serotype 2 was reported in Taiwan, and serotype 3 was first characterised in South Korea and China [2,3]. The evolution of the DHAV is driven by the accumulation of point mutations and/or recombination between different serotypes [4]. 

DHAV has a single-strand positive-sense RNA genome of ~7.8 kb size that encodes a single viral polyprotein (VP) flanked with untranslated regions (UTR) at the 5′ and 3′ ends [5,6]. The 5′ UTR has a distinctive internal ribosome entry site (IRES), which is essential for initiating translation and virus RNA synthesis. The VP polyprotein is ~2200 amino acids in length and is spliced into three structural proteins (VP0, VP1 and VP3) and nine non-structural proteins (2A1, 2A2, 2A3, 2B, 2C, 3A, 3B, 3C and 3D/RNA-dependent RNA-polymerase). VP0 is further subdivided into VP2 and VP4. The VP1 protein is highly variable [6,7,8] and plays a major role in receptor binding, virulence and immunogenicity [9]. 

DHAV was reported in Egypt in the late 1970s [10], and later, several reports of the disease outbreaks have been documented from Egypt [11,12,13]. These reports mainly focused on partial sequencing of the 5′UTR, VP1 and 3D genes and thus failed to provide a comprehensive genome-wide evolutionary insight. Since the expansion of the Egyptian duck farms, DHAV has caused devastating losses and poses a major threat to the commercial duck farms [11,12,13]. To effectively control the disease, mass vaccination programmes have been implemented in Egypt through vaccination of breeder ducks using attenuated vaccines produced from the E52 Rispens strain [11,14]. Therefore, it is of paramount importance to establish epidemiological insights into the virus evolution to identify risks and challenges. The aim of the current study is to investigate the prevalence of DHAV in ducklings with a history of high mortality in different Egyptian governorates between 2018– 2020 based on virus isolation and complete genome sequencing.

## 2. Materials and Methods

### 2.1. Ethics Statement

Samples were collected by trained veterinarians from duckling flocks kept as commercial livestock in farms. Tissue samples (liver and spleen) were obtained from dead ducklings, which did not require any anaesthesia. Samples processing and virus isolation procedures were carried out in strict accordance with the guidance and regulations of animal welfare and health that approved by the Ethics Committee at Veterinary Serum and Vaccine Research Institute (VSVRI), Agricultural Research Centre (ARC), Cairo, Egypt.

### 2.2. Samples Collection, Virus Detection and Isolation

Eighteen samples (liver and spleen) were collected between 2018–2020 from different commercial 3–11-day-old duckling flocks (Pekin and Mullard) that had a history of nervous signs and high mortalities within six Egyptian governorates (Table 1). Liver and spleen samples from each farm/flock were pooled and treated as one sample/farm. All samples were stored at −80 °C until processing. Viral RNA was extracted from the supernatant of tissue homogenates using the QIAamp Viral RNA Mini kit (Qiagen, GmbH, Hilden, Germany) according to the manufacturer’s instructions. RT-PCR assay was conducted to detect DHAV through partial amplification of the VP1 gene [9]. Virus isolation was carried out for the RT-PCR-positive samples by inoculation into the allantoic cavity of 10 to 14-day-old embryonated duck embryos (EDEs) according to the standard protocols [15] for three consecutive passages. Eggs were candled daily for up to 7 days, and embryos were examined for pathologic lesions, including stunting, oedema and/or haemorrhages, particularly in the liver, kidneys and spleen [15].

### 2.3. Complete Genome Sequencing

Total RNA was extracted from the allantoic fluid using TRIzol^TM^ reagent (Invitrogen, Waltham, MA, USA) according to the manufacturer’s instructions. The concentration and purity of extracted RNA were measured using a spectrophotometer, ND-1000 (Nanodrop Technologies, Wilmington, DE, USA), and the integrity of RNA was visualised by electrophoresis in a 1.2% formaldehyde agarose gel stained with GelRed^®^ (Biotium, Fremont, CA, USA). The extracted RNA was kept at −80 °C until use. Complete genome sequencing was carried out as previously described [16]. In order to amplify nine overlapped PCR fragments, nine pairs of specific primers were used. A gel purification kit (Invitrogen, Waltham, MA, USA) was used to purify the PCR products. These products were then TA-cloned into the pMD18-T vector (Takazawa, Tokyo, Japan) according to the manufacturer’s protocol. The TA-cloned products were transformed into *E. coli* strain DH5α cells (Invitrogen, Waltham, MA, USA). The positive clones were selected by PCR and sequenced with the dideoxy terminal termination method using Applied Biosystems genetic analyser 3500xl (ThermoFisher, Waltham, MA, USA) by Source Bioscience Co., Ltd., UK.

### 2.4. Genetic and Phylogenetic Analyses

The obtained nucleotide sequences were aligned using MUSCLE v. 3.8.31 [17], analysed with DNAstar Lasergene (version 13) programs and submitted to GenBank database under the following accession numbers MZ004919-MZ004923. The nucleotide pairwise identity scores were predicted using the Sequence Demarcation Tool (SDT) through a colour-coded matrix [18]. Phylogenetic analyses were conducted using Molecular Evolutionary Genetics Analysis (MEGA) version 6.0 [19]. The phylogenetic trees were constructed using the Maximum Likelihood (ML) method using RaxML version 8.2.11 [20], and the general time-reversible (GTR) model of nucleotide substitution with gamma-distributed rate variation among the sites was selected based on the jModelTest [21]. 

### 2.5. Detection of Putative Recombination and Selective Pressure

The Recombination Detection Program 4 (RDP version 4.97) software has been used to detect potential recombination events in the DHAV complete genomes [22] through various program detection methods (RDP, Genecov, Bootscan, Maxchi, Chimaera, Siscan and 3Seq) with a modified *p*-value of 0.05. Only recombination with no less than five independent methods was considered optimistic. The Synonymous-Non-Synonymous Analysis Program (SNAP) was used to predict the VP1 gene-specific estimates of dN/dS [23]. The number of potential synonymous and non-synonymous changes were counted as well as the number of actual synonymous and non-synonymous changes in codon between each pair. The dN/dS ratio was calculated by comparing the proportion of observed non-synonymous substitutions over the proportion of observed synonymous substitutions. These were then adjusted for multiple hits using the Jukes–Cantor correction [23].

### 2.6. Histopathology

Liver samples were collected, fixed in neutral buffered formalin 10%, washed, dehydrated, cleared and embedded in paraffin [24]. The paraffin-embedded blocks were sectioned at 5 µm thickness and stained with Haematoxylin and Eosin for light microscopic examination, Olympus BX50, Tokyo, Japan. Semiquantitative histopathologic scoring was carried out according to a modified scoring system [13] for microscopic evaluation of hepatic tissue damage on a scale from 0 to 3 based on the lesion severity grade (mild, moderate, severe) as follows: 0 = no changes, 1 = mild, 2 = moderate and 3 = severe. Briefly, the assigned alterations (five parameters) were congestion, haemorrhage, vacuolar degeneration of hepatocytes, hepatocellular apoptosis and inflammatory cells infiltration.

## 3. Results

### 3.1. Virus Screening and Isolation

All samples were collected from ducklings showing lethargic, nervous signs of ataxia and a high mortality rate (~60%). Upon necropsy, livers and spleens were enlarged and haemorrhagic, petechial or ecchymotic. RT-PCR was carried out for the screening of DHAV-1 based on the VP1 gene. Five samples were positive out of eighteen (5/18; 27.8%) (Table 1). The Pekin duckling breed showed a higher infection rate (4/5; 80%) compared to the Mallard breed (1/5; 20%) (Table 1). The RT-PCR positive samples (*n* = 5) were subjected to virus isolation on EDEs for three passages and observed embryonic deaths within 5–7 days post-inoculation. Embryonic examination showed oedema with abdominal distension, cutaneous haemorrhages, stunting or dwarfing, hepatitis and enlarged congested kidneys and spleens on the second passage. The harvested allantoic fluid was negative HA to exclude the chance of any mixed infection with hemagglutinating viruses (Newcastle disease virus or avian influenza viruses: H5N1, H5N8 and H9N2 or duck adenovirus 1), which are endemic in Egypt.

### 3.2. Genetic Characterisation of Complete Genomes

Genetic recombination and point mutations are the main driving forces of DHAV evolution [4]. Unfortunately, due to the unavailability of any complete genome sequences for Egyptian DHAV-1 viruses on databases, it was difficult to compare the whole genome. The studied DHAV-1 isolates’ complete genomic length is around 7,691 nucleotides (nt), with the exclusion of a 17-nt poly (A) 3′ end tail. A single open reading frame (ORF) has a size of 6,750 nt, encodes a putative polyprotein precursor of 2,249 amino acids, flanked by a 627-nt (UTR) region, and a 315-nt 3′ UTR. The process of cleavage was observed in a total of 11 cleavage sites, which lead to the generation of 12 proteins, VP0, VP3, VP1, 2A1, 2A2, 2A3, 2B, 2C, 3A, 3B, 3C and 3D.

**Figure 1:**
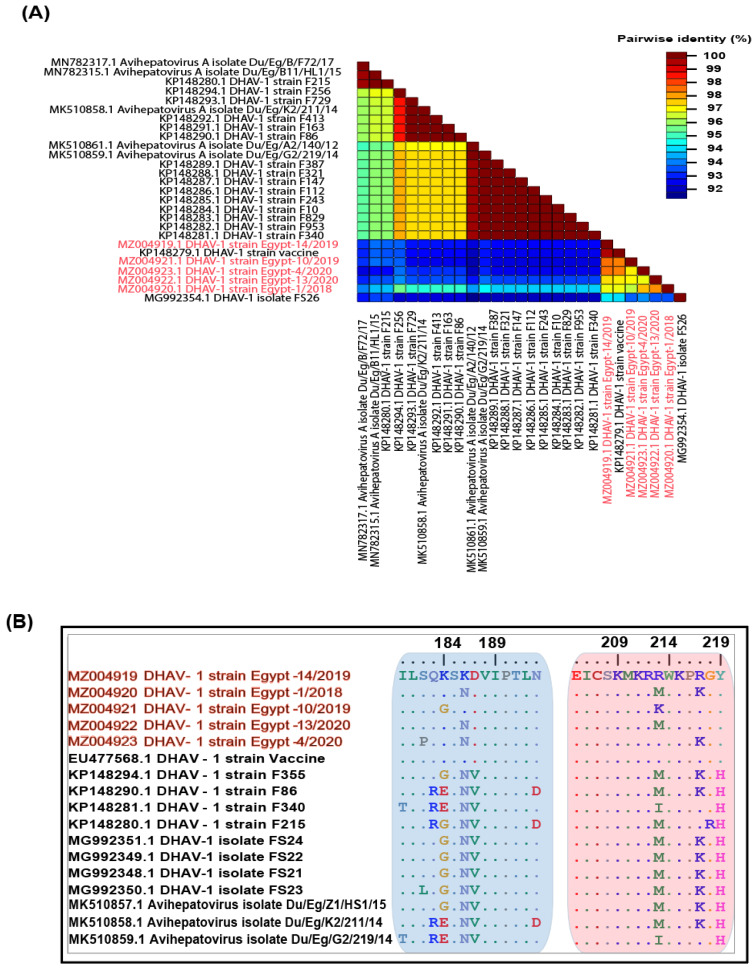
The pairwise identities plot of VP1 gene for DHAV-1 isolates reported in this study (red coloured) aligned by ClustalW and displayed by (**A**) Sequence Demarcation Tool (SDT) software and (**B**) localisation of specific mutations in the hypervariable areas (aa 180–193 and aa 205–219) within the VP1 protein of the newly identified DHAV-1 strains.

Interestingly, the nucleotide identity matrix for the VP1 gene of our reported isolates ranged from 92% to 95% compared to the previously reported Egyptian field strains, while 96% to 98% with the vaccine strain (Figure 1A), suggesting that these strains might be driven from the DHAV-1 vaccine and or due to the role of vaccination pressure over the field strains. On the other hand, there are three hypervariable regions (HVRs) at the VP1 protein C-terminal amongst DHAV-1 strains that showed a high level of genetic diversity [7]. In this study, two hypervariable areas (aa 180–193 and aa 205–219) revealed marked amino acids substitutions compared to the previously reported DHAV-1 field and vaccine strains. The reported isolates possessed S182P (DHAV-1 strain Egypt-4/2020), G/E184K (DHAV-1 strain Egypt-14/2019, DHAV-1 strain Egypt-1/2018, DHAV-1 strain Egypt-13/2020 and DHAV-1 strain Egypt-4/2020), N186K (DHAV-1 strain Egypt-14/2019 and DHAV-1 strain Egypt-10/2019) and V187D mutations in the carboxy terminal region (Figure 1B). Other mutations were observed in the VP1 protein, as shown in Figure S1.

### 3.3. Phylogenetic Analyses

**Figure 2:**
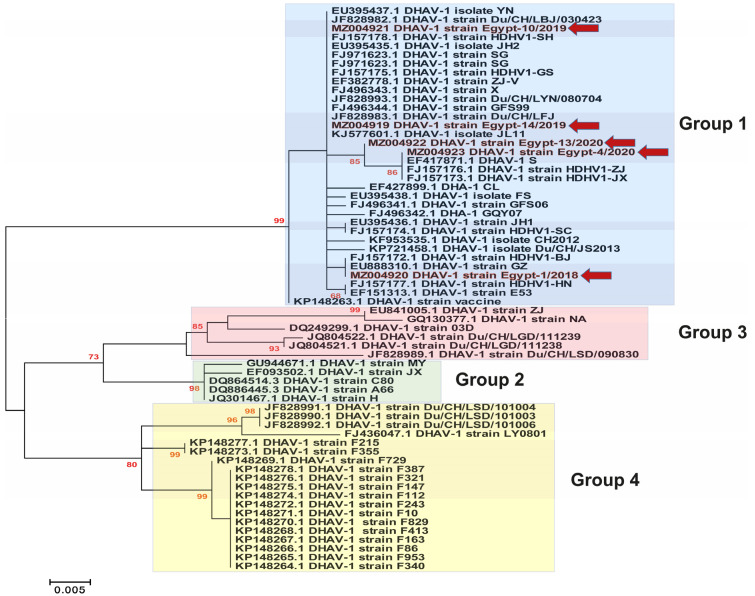
Phylogenetic analyses based on the 3D gene of the newly identified DHAV-1 strains (highlighted with red colour along with red arrows) in Egypt between 2018–2020 showed the clustering pattern for the studied DHAV-1 strains. DHAV-1, genotype I is divided into four groups (1–4) that are highlighted by colour boxes as follows: group 1 (light blue), group 2 (light green), group 3 (light pink) and group 4 (light yellow). The phylogenetic trees were constructed using the Maximum Likelihood (ML) method using RaxML version 8.2.11, and the general time-reversible (GTR) model of nucleotide substitution with gamma-distributed rate variation among sites was selected based on the jModelTest. Bootstrap values (>60%) are indicated above the branches of the tree.

**Figure 3:**
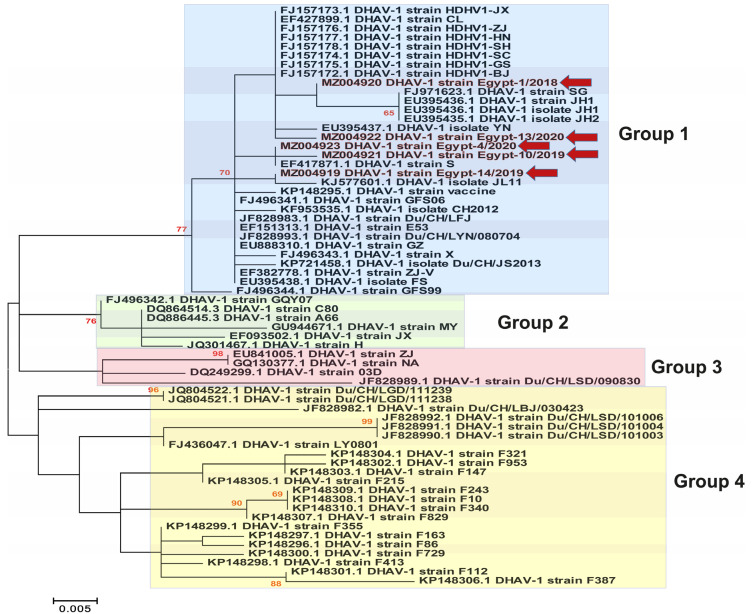
Phylogenetic analyses based on the 5′UTR gene of the newly identified DHAV-1 strains (highlighted with red colour along with red arrows) in Egypt between 2018–2020 showed the clustering pattern for the studied DHAV-1 strains. DHAV-1, genotype I is divided into four groups (1–4) that are highlighted by colour boxes as follows: group 1 (light blue), group 2 (light green), group 3 (light pink) and group 4 (light yellow). The phylogenetic trees were constructed using the Maximum Likelihood (ML) method using RaxML version 8.2.11, and the general time-reversible (GTR) model of nucleotide substitution with gamma-distributed rate variation among sites was selected based on the jModelTest. Bootstrap values (>60%) are indicated above the branches of the tree.

**Figure 4:**
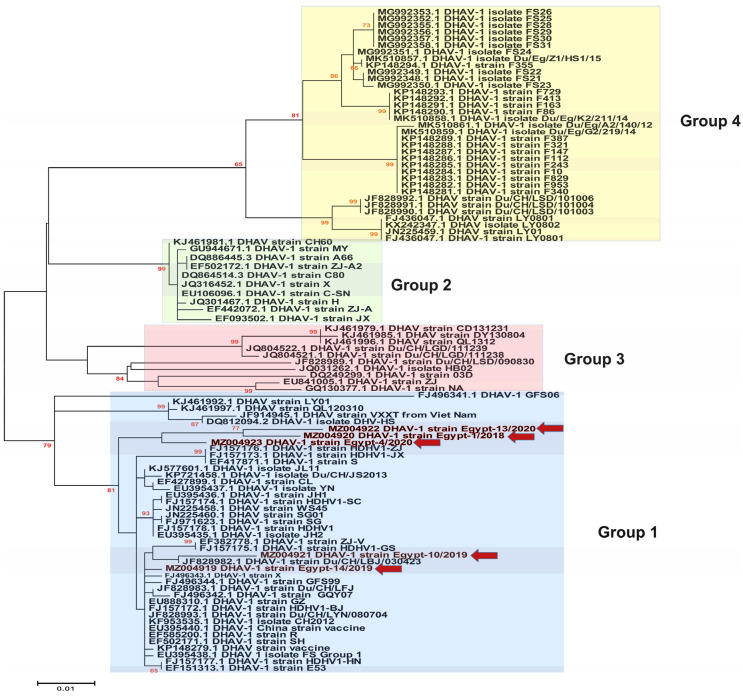
Phylogenetic analyses based on the VP1 gene of the newly identified DHAV-1 strains (highlighted with red colour along with red arrows) in Egypt between 2018–2020 showed the clustering pattern for the studied DHAV-1 strains. DHAV-1, genotype I is divided into four groups (1–4) that are highlighted by colour boxes as follow; group 1 (light blue), group 2 (light green), group 3 (light pink) and group 4 (light yellow). The phylogenetic trees were constructed using the Maximum Likelihood (ML) method using RaxML version 8.2.11, and the general time-reversible (GTR) model of nucleotide substitution with gamma-distributed rate variation among sites was selected based on the jModelTest. Bootstrap values (>60%) are indicated above the branches of the tree.

**Figure 5:**
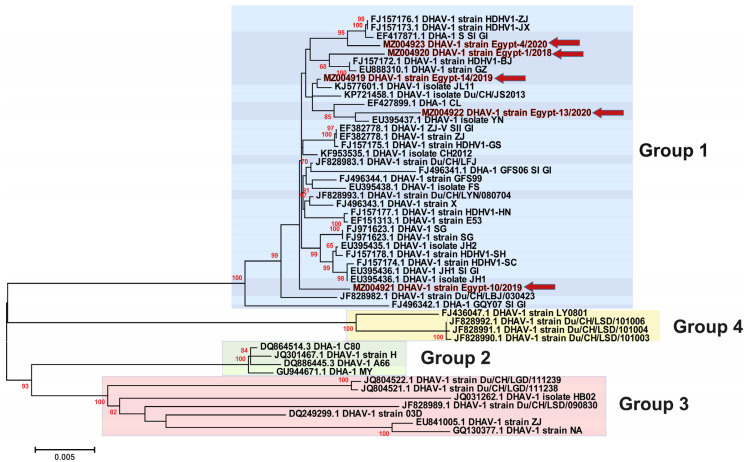
Phylogenetic analyses based on the complete genome of the newly identified DHAV-1 strains (highlighted with red colour along with red arrows) in Egypt between 2018–2020 showed the clustering pattern for the studied DHAV-1 strains. DHAV-1, genotype I is divided into four groups (1–4) that are highlighted by colour boxes as follows: group 1 (light blue), group 2 (light green), group 3 (light pink) and group 4 (light yellow). The phylogenetic trees were constructed using the Maximum Likelihood (ML) method using RaxML version 8.2.11, and the general time-reversible (GTR) model of nucleotide substitution with gamma-distributed rate variation among sites was selected based on the jModelTest. Bootstrap values (>60%) are indicated above the branches of the tree.

Previous studies have classified the DHAV-1 isolates into four major genogroups/genotypes (GI, GII, GIII and GIV) based on the phylogenetic analysis of the VP1 gene [7]. In the current study, we selected representative strains for GI to be included within all the phylogenetic analyses, especially due to the high similarity between the previously reported Egyptian DHAV-1 strains and the Chinese strains of GI. Phylogenetic analysis revealed that most of the Egyptian DHAV-1 isolates are clustered within group 4 of DHAV-1 genotype I, while our five isolates reported in this study were clustered within group 1 based on the 3D gene (Figure 2), 5′UTR (Figure 3), VP1 gene (Figure 4) and complete genome (Figure 5). Interestingly, most of the previously characterised Egyptian DHAV-1 strains were clustered within group 4, where all characterised isolates in this study were clustered within group 1 that were subdivided into at least three subgroups. Due to unavailability for the Egyptian complete genome sequences, we tried to compare between the phylogenetic trees based on either the 3D (Figure S2), 5′UTR (Figure S3) or VP1 (Figure S4) genes and the complete genome-based phylogeny to have the closest model to construct the evolutionary trees based on which gene and to compute a consensus tree in the context of bootstrap analysis.

### 3.4. Recombination and Selective Pressure Analyses

**Figure 6:**
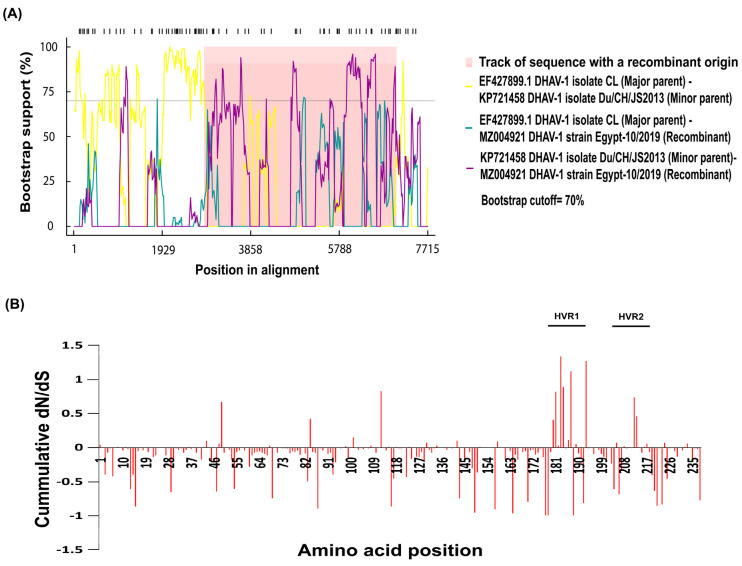
Recombination and selective pressure analyses. (**A**) Recombination detection analysis displaying possible recombination events predicted within the VP1 gene of the Egyptian DHAV-1 strains. (**B**) The cumulative dN/dS of the average synonymous and non-synonymous substitutions moving codon by codon across VP1 gene.

Recombination is an important approach to achieve genetic diversity and is recognised as an important part of the viruses’ evolution. Recombinant strains could have distinct biological characteristics and yield different clinical forms compared to parental strains. However, the molecular understanding of recombination of DHAV-1 is little known. Therefore, the analysis of the recombination events in DHAV-1 could provide valuable information for the understanding of the DHAV-1 evolution. Our findings showed the emergence of one new recombinant virus, with a high score *p* < 0.01 and a recombinant score >0.6. The recombinant virus (DHAV-1 strain Egypt-10/2019) could emerge as a result of recombination between EF427899.1 DHAV-1 isolate CL (major parent) and KP721458 DHAV-1 isolate Du/CH/JS2013 (minor parent) (Figure 6A), both are of Chinese origin (Asian lineage) that is closely related to the Egyptian viruses. In addition, the recombination events have been confirmed with the phylogeny based on the region included within recombination within major and minor parents (Figure S5). 

The uniform differences in dN-dS were estimated for each position to further determine the existence of a differential selective pressure intensity on the VP1 protein. Scores above 0 indicate a greater diversification of selection, while this value was also used for the measurement of a cumulative score by summing codon per codon. A pairwise comparison bioinformatics approach (SNAP) was applied to determine the synonymous and non-synonymous substitution rates and selective evolutionary pressure for the VP1 protein. The selection profiles of the amino acid sequence for all the five Egyptian DHAV-1 strains showed different patterns within the VP1 protein numbers above zero indicate the positive selection, around zero shows the neutral selection, and below zero indicates the negative or purifying selection (Figure 6B). 

### 3.5. Histological Examinations

**Figure 7:**
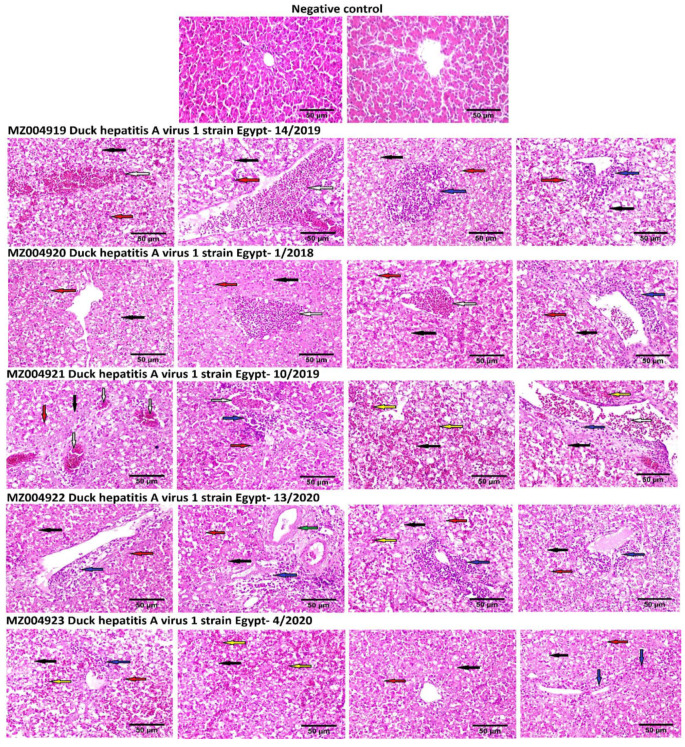
Photomicrographs of Haematoxylin and Eosin-stained liver sections of negative control (non-infected) compared to DHAV-1-infected ducklings showing severe dilatation and congestion of the central vein (white arrow), vacuolar degeneration of hepatocytes (black arrow), marked hepatocellular apoptosis (red arrow), inflammatory cells infiltration, mononuclear and heterophils (blue arrow), haemorrhage (yellow arrow) and hyperplasia of the biliary epithelium (green arrow). Non-infected (negative control) ducklings showed normal liver. Scale bar shows 50 μm.

Light microscopic examination for the liver of non-infected (negative control) ducklings compared to DHAV-infected ducklings revealed remarkable histopathological alterations exhibited by severe dilatation and congestion of central veins and hepatic sinusoids, marked vacuolar degeneration of hepatocytes and hepatocellular necrosis associated with marked hepatocellular apoptosis. The portal triad showed hyperplasia of biliary epithelium, portal infiltration with mononuclear cells, mainly lymphocytes and heterophils. Additionally, perivascular inflammatory cells infiltration (lymphocytes and heterophils) and focal hepatic haemorrhage were also seen in the examined sections. (Figure 7). The recombinant strain (DHAV-1 strain Egypt-10/2019) showed significant histopathological alterations compared to normal (negative control) and other DHAV-1 strains identified in this study (Table S1).

## 4. Discussion

Despite DHAV causes devastating losses in the duck industry for decades within the African and Middle East countries, including Egypt, there is little information available on the disease prevalence. The high mortality rate associated with the DHAV-1 outbreaks, which is often over 50% and can exceed up to 95% in field conditions, continues to be a threat to duck farms [12,25]. The infection severity for young ducklings with DHAV is mainly age-dependent due to their immune system immaturity, which is unable to protect them from virus infection and replication [26]. Previous studies have reported that Muscovy ducklings were stable carriers for DHAV-1 infections, while Pekin, Mallard and hybrid breeds infections are associated with acute disease. Furthermore, Muscovy ducks are genetically distinct and form a distinct genetic group away from other duck breeds [27]. However, this genetic variation remains unclear for the susceptibility to DHAV-1 infection. Of note, pancreatitis and encephalitis have been associated with recent variant strains belonging to DHAV genotype 3 and revealed 25%–40% deaths without serious liver lesions [28,29]. 

In this study, DHAV was screened in suspected duck farms in six of Egypt’s provinces. The majority of the examined field outbreaks were in ducklings aged 3–11 days old. The collected sample homogenate was screened based on partial VP1 gene amplification and RT-PCR testing revealed five positive samples. These positive samples were isolated in 10–14-day EDEs. Pathogens other than DHAV, such as Pasteurella, Salmonella or E. coli, could be responsible for a high mortality rate in ducklings, particularly in DHAV-negative samples. In this study, we tried to allocate the majority of available DHAV-1 strains on databases to have a genotyping-based classification system based on complete genome, VP1, 3D and 5′UTR. Deep genetic analyses showed that the C-terminal of the VP1 protein of DHAV-1 carried two hypervariable regions (HVRs); HVR1 (180–194) and HVR2 (205–219) that have been correlated with DHAV-1 virulence differences [9,30]. Besides that, Wang et al. [6] have shown that Asian virulent and attenuated DHAV-1 strains have similar VP1 sequences with minor differences in the VP0 and VP3. This suggests that DHAV-1 virulence may be correlated with other genomic regions other than VP1 protein. Most of the previous studies have classified the DHAV based on the antigenic and/or neutralisation characteristics that classified DHAV-1 strains into four main groups (1–4) [13,25]. The Egyptian isolates were clustered along with Chinese virulent viruses’ group 4 that has been subgrouped into A, B1, B2 and C according to their geographic distribution [13,25]. Putative amino acids revealed distinctively marked amino acids substitutions within two hypervariable regions (aa 180–193 and aa 205–219) compared to the vaccine strains being used in the Egyptian industry. Our isolates carried S182P (DHAV-1 strain Egypt-4/2020), G/E184K (DHAV-1 strain Egypt-14/2019, DHAV-1 strain Egypt-1/2018, DHAV-1 strain Egypt-13/2020 and DHAV-1 strain Egypt-4/2020), N186K (DHAV-1 strain Egypt-14/2019 and DHAV-1 strain Egypt-10/2019) and V187D mutations in the carboxy terminal region.

Phylogenetic analyses revealed that DHAVs genotype I was diversified into four major groups; 1–4, where our isolates were clustered within group 1. In addition, sequencing and phylogenetic analyses indicated that the Egyptian field strains are distinguishable from the commercially used vaccine strain where lower identity was observed in VP1. The VP1 is the most external surface protein and is involved in receptor binding and contains neutralising epitopes [9]. Likewise, it is highly recommended to update the vaccines according to the circulating viruses being reported from outbreaks in ducklings [3,31]. Interestingly, the DHAV-1 strain Egypt-10/2019 (recombinant strain) exhibited significant histopathological alterations compared to normal (negative control) and other DHAV-1 strains identified in this study, while no significant differences were identified in the mortality rates of affected duckling flocks.

In conclusion, it is unclear whether the commercially available vaccine is not able to protect ducklings from the field strains due to antigenic and or genetic differences. Matching studies on the antigenic and genetic relationship between these strains are also required. In addition, further research is needed to catalogue various DHAV strains based on the proposed virulence markers. Meanwhile, in vivo testing of the commercial DHAV vaccines currently used are important to demonstrate their efficacy in battling DHAV strains and to investigate the pathobiology of newly evolving DHAV-1 strains, which can aid in exploring their pathogenesis and monitoring their developmental changes. Likewise, the genetic variations between recent Egyptian strains and commercial vaccines are urging the efficacy assessment and/or production of new vaccine candidates. Finally, studying the relationship between DHAV-1 infection susceptibility and biodiversity of duck breeds will lead to a better understanding of the pathogenic heterogeneity of various DHAV-1 strains.

## Figures and Tables

**Table 1:** Data of collected samples for duck hepatitis A virus screening from 2018 to 2020 in Egypt.

Sample ID	Breed	Age (Days)	Governorate	Year	Virus Detection (RT-PCR)	Strain
1	Pekin	5	Giza	2018	Yes	DHAV-1 strain Egypt-1/2018
2	Pekin	4	Monufia	2018	-	-
3	Mallard	6	Qalyubia	2018	-	-
4	Mallard	5	Monufia	2020	Yes	DHAV-1 strain Egypt-4/2020
5	Pekin	7	Qalyubia	2019	-	-
6	Mallard	6	Giza	2019	-	-
7	Mallard	9	Faiyum	2018	-	-
8	Pekin	8	Giza	2019	-	-
9	Mallard	10	Gharbia	2019	-	-
10	Pekin	4	Giza	2019	Yes	DHAV-1 strain Egypt-10/2019
11	Pekin	3	Giza	2020	-	-
12	Mallard	10	Monufia	2019	-	-
13	Pekin	4	Beni Suef	2020	Yes	DHAV-1 strain Egypt-13/2020
14	Pekin	4	Faiyum	2019	Yes	DHAV-1 strain Egypt-14/2019
15	Mallard	9	Beni Suef	2019	-	-
16	Mallard	3	Giza	2020	-	-
17	Pekin	8	Qalyubia	2019	-	-
18	Mallard	5	Faiyum	2020	-	-

-: means negative.

## Data Availability

Not applicable.

## Supplementary Materials

The following are available online at https://www.mdpi.com/article/10.3390/ani11092741/s1, Figure S1: Deduced amino acid identity shows the mutations within VP1 protein for our five isolates reported in this study compared to previously reported Egyptian isolates and vaccine strain. Figure S2: Phylogenetic analyses based on the 3D gene (a) compared with the complete genome (b) for the newly identified DHAV-1 strains (highlighted with red colour along with red arrows) in Egypt between 2018–2020 showed the clustering pattern for the studied DHAV-1 strains within genotype I that is divided into four groups (1–4) and highlighted by colour boxes as follows: group 1 (light blue), group 2 (light green), group 3 (light pink) and group 4 (light yellow). Figure S3: Phylogenetic analyses based on 5′UTR gene (a) compared with the complete genome (b) for the newly identified DHAV-1 strains (highlighted with red colour along with red arrows) in Egypt between 2018–2020 showed the clustering pattern for the studied DHAV-1 strains within genotype I that is divided into four groups (1–4) and highlighted by colour boxes as follows: group 1 (light blue), group 2 (light green), group 3 (light pink) and group 4 (light yellow). Figure S4: Phylogenetic analyses based on VP1 gene (a) compared with the complete genome (b) for the newly identified DHAV-1 strains (highlighted with red colour along with red arrows) in Egypt between 2018–2020 showed the clustering pattern for the studied DHAV-1 strains within genotype I that is divided into four groups (1–4) and highlighted by colour boxes as follows: group 1 (light blue), group 2 (light green), group 3 (light pink) and group 4 (light yellow). Figure S5: Neighbour joining tree of region derived from A) a major parent and B) a minor parent for DHAV-1 strain Egypt-10/2019 (recombinant strain). Table S1. Histopathologic lesion scores in the different DHAV-1 isolates reported in this study.
