# Supplementary material for: Insights into the Genetic Evolution of Duck Hepatitis A Virus in Egypt
Source: Animals (Basel). 2021 Sep 19;11(9):2741. doi: 10.3390/ani11092741 (PMC8472559; doi:10.3390/ani11092741)
Supplement: Supplementary file 1 [file animals-11-02741-s001.zip › Table S1 lesion scores.pdf]

Table S1. Histopathologic lesion scores in the different DHAV-1 isolates reported in this study.

Histopathological lesion	Normal control	DHA virus 1 strain Egypt-14/2019	DHA virus 1 strain Egypt-1/2018	DHA virus 1 strain Egypt-10/2019	DHA virus 1 strain Egypt-13/2020	A virus 1 strain Egypt-4/2020
**Congestion of central vein**	0.0 ± 0.0	2.6 ± 0.5	2.2 ± 0.4	3 ± 0.0	1.6 ± 0.5	1.4 ± 0.5
**Hemorrhage**	0.0 ± 0.0	1.4 ± 0.5	0.6 ± 0.5	2.6 ± 0.5	1.2 ± 0.4	1.6 ± 0.5
**Vacuolar degeneration of hepatocytes**	0.0 ± 0.0	2.6 ± 0.5	2.2 ± 0.4	3 ± 0.0	2.2 ± 0.4	2.4 ± 0.5
**Hepatocellular apoptosis**	0.0 ± 0.0	2.4 ± 0.54	2.4 ± 0.5	3 ± 0.0	2.2 ± 0.8	1.6 ± 0.5
**Inflammatory cells infiltration**	0.0 ± 0.0	1.4 ± 0.5	1.6 ± 0.5	2.8 ± 0.4	2 ± 0.7	1.6 ± 0.5

Data shown as mean ± SD. One–way analysis of variance was used for data analysis, significantly different ($p < 0.05$).
