# Supplementary figures and images for: Insights into the Genetic Evolution of Duck Hepatitis A Virus in Egypt
Source: Animals (Basel). 2021 Sep 19;11(9):2741. doi: 10.3390/ani11092741 (PMC8472559; doi:10.3390/ani11092741)

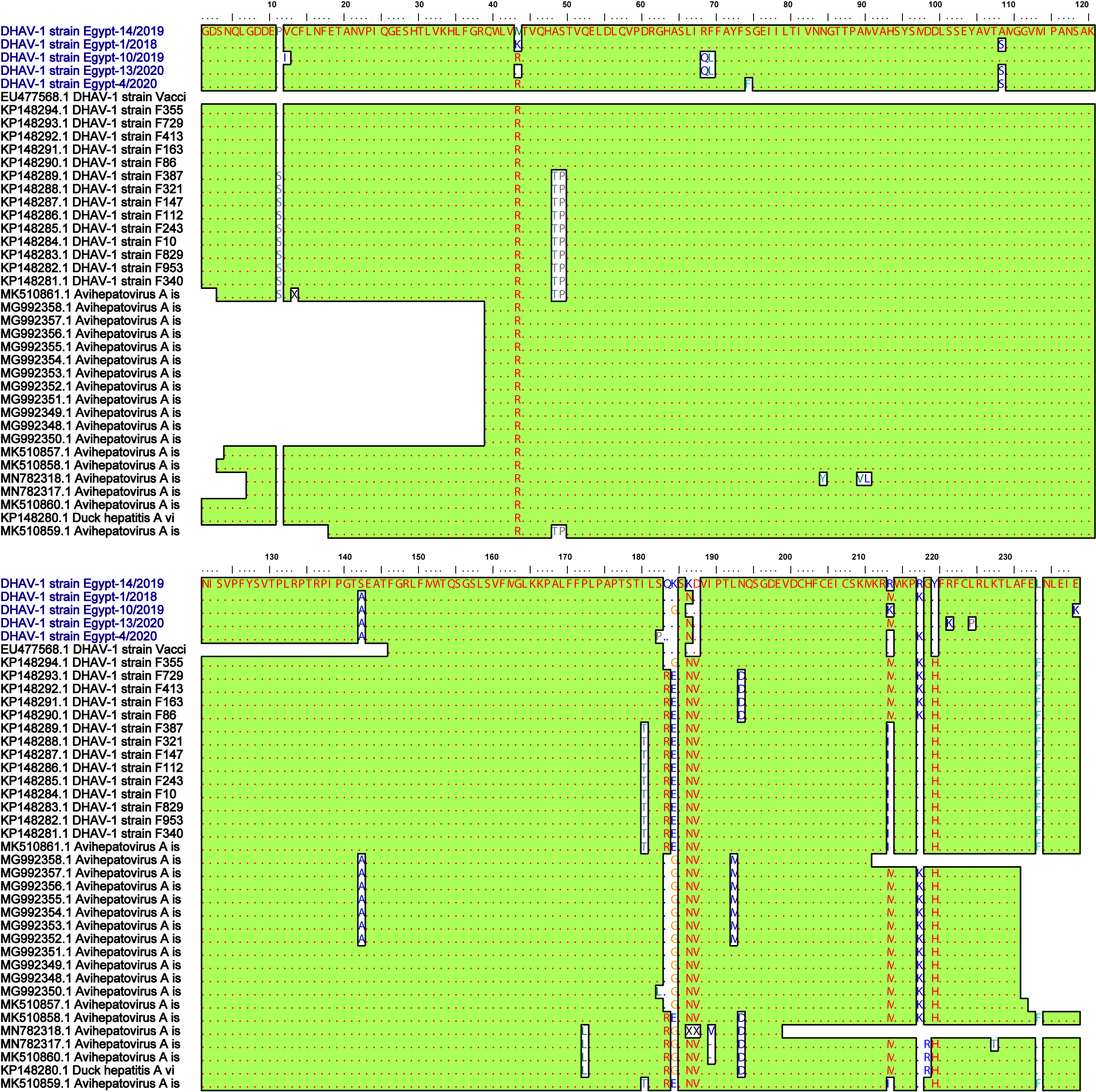

Supplement: Supplementary file 1 [file animals-11-02741-s001.zip › Suplementary figure S1_amino acids mutation VP1.tif]
